# Imaging and simulation study of electrokinetic supercharging in flow-gated capillary electrophoresis

**DOI:** 10.1007/s00216-025-06116-3

**Published:** 2025-09-20

**Authors:** Ying Gong, Zhengyang Ye, Atena Tajaddodi, Maojun Gong

**Affiliations:** Department of Chemistry and Biochemistry, Wichita State University, 1845 Fairmount St, Wichita, KS 67260 USA

**Keywords:** Electrokinetic supercharging, Isotachophoresis, FASI, Flow-gated CE, Fluorescence imaging, Glyphosate

## Abstract

Electrokinetic supercharging (EKS), an efficient sample preconcentration technique, has demonstrated millionfold concentration enhancement. Despite extensive studies, the detailed behavior of associated ions during EKS remains insufficiently understood. This research aims to elucidate the underlying EKS mechanisms on a flow-gated capillary electrophoresis (CE) system by using the combination of fluorescence imaging, computer simulations, and comparative analysis of electropherograms under various pairs of leading/terminating electrolytes (LE/TE). Two distinct operational modes were evaluated to highlight procedural differences: simultaneous EKS and sequential EKS. In the simultaneous mode, transient isotachophoresis (tITP) was conducted concurrently with field-amplified sample injection (FASI). For effective operation, LE anions were included in background electrolyte (BGE), while the TE anionic plug was generated by FASI from TE anions included in the sample. In the sequential mode, FASI was performed first to inject and preconcentrate analytes along with LE anions present in the sample, and the preconcentrated LE plug then functioned as LE in the subsequent tITP stage. While sequential EKS generally yielded lower signal enhancement compared to the simultaneous approach, it offered improved operational control and reproducibility. Due to its greater robustness, sequential EKS was chosen for the determination of herbicide residues in cereal samples. This study offers detailed insights into ion dynamics under various EKS configurations and provides practical guidelines for selecting LE/TE compositions and optimizing buffer conditions in flow-gated CE systems.

**Graphical abstract:**

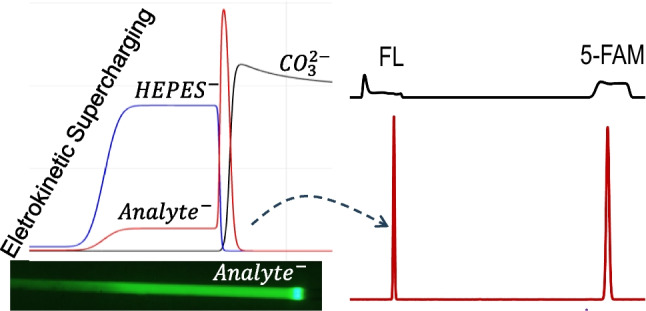

**Supplementary Information:**

The online version contains supplementary material available at 10.1007/s00216-025-06116-3.

## Introduction

Electrokinetic supercharging (EKS) is a sample preconcentration technique that integrates electrokinetic injection with transient isotachophoresis (tITP) [1]. When coupled with field-amplified stacking injection (FASI), the resulting FASI-EKS method can rapidly inject target analytes and significantly enhance the detection sensitivity [2, 3]. In conventional capillary electrophoresis (CE), this approach can easily yield detection enhancement factors of a few thousand [4–6]. To further improve the stacking effectiveness, a counter-flow has been employed to maintain a stationary boundary for extended injections, and thus up to 100,000 folds of signal enhancement have been observed [7–9]. Moreover, EKS has achieved a millionfold concentration increase on a simple cross glass microchip under conditions of electroosmotic flow (EOF) suppression and high leading electrolyte concentration [10].

Transient ITP plays a crucial role in the stacking mechanism of EKS [11]. tITP is generally categorized into two modes: medium-induced tITP and sample-induced tITP [2]. In medium-induced tITP, a sample plug is sandwiched between a terminating electrolyte (TE) and a leading electrolyte (LE). The LE and TE can be introduced as individual plugs or included as co-ions in background electrolytes (BGE). In contrast, sample-induced tITP involves mixing either or both LE and TE ions into the sample matrix, while any remaining LE or TE components can be included in the BGE [12, 13]. In conventional CE, LE and TE plugs are usually introduced sequentially into the capillary before and after the sample plug, respectively, resulting in a double-sandwich configuration: BGE, TE, analytes, LE, and BGE [11, 14]. However, implementing this configuration becomes more complex in microchip or flow-gated CE, where BGE or sample solutions are delivered through fixed reservoirs or continuous flows rather than movable vials on a rack, as in a conventional CE system [15, 16]. Therefore, intricate microfluidic networks or flow control designs are required to achieve the double-sandwich configuration for tITP [11]. To simplify operation, LE and TE ions are frequently incorporated into buffer solutions or used as buffer components for tITP. For example, Jeong et al. used Cl^−^ existing in the saline sample matrix as LE, mixed TE ions of N-tris(hydroxymethyl)-methyl-3-aminopropane-sulfonic acid (TAPS) in BGE, and concentrated model analytes by 500 folds on a microchip with a long serpentine injection channel [17]. Similarly, Jung et al. utilized HEPES anions as TE in the sample solution and Cl^−^ as LE in BGE, and achieved significant signal enhancement in a simple cross microchip [10].

FASI can rapidly introduce a large amount of analytes into a capillary, effectively overcoming the volume limitations typically associated with CE [18]. However, the injected analyte plugs often require further compression to preserve separation efficiency [19, 20]. As a robust and effective stacking method, tITP can be combined with FASI to remarkably enhance performance. In the experimental operation of EKS, FASI and tITP can be conducted simultaneously for an elongated time. Afterwards, the sample solution is replaced with BGE for further tITP stacking before separations [10, 21, 22]. For simultaneous FASI-EKS, a high-mobility anion such as chloride present in BGE serves as LE while a low-mobility anion such as HEPES in the sample serves as TE [10]. To sustain stacking after FASI, the voltage polarity is usually maintained. This scenario has been discussed in detail by Dr. Breadmore and Dr. Quirino in 2008, where a concentration enhancement factor of up to 100 K was obtained for anions when a counterflow was used to stabilize the ITP stacking boundary [7].

To adapt EKS to flow-gated CE, a procedure has been developed to electrokinetically introduce a low-conductivity plug followed by FASI under a reversed-polarity voltage [23]. This approach achieved 800-fold enhancement in detection sensitivity. Different from the simultaneous FASI-EKS, the method first uses FASI to rapidly inject and concentrate anionic analytes along with LE anions at the conductivity boundary. The resulting analyte plug is then further focused via tITP.

This research aims to perform a comprehensive study of FASI-EKS mechanisms in flow-gated CE with polarity switching. Electropherograms were recorded under various LE/TE pairs to examine the stacking conditions. Additionally, fluorescence imaging was used to visualize the migration behavior of anionic analytes, while computer simulations were conducted to model the dynamics of the associated ions during FASI and tITP processes. Finally, the sequential EKS method was employed to determine glyphosate residues in cereal samples. These results are expected to provide practical guidelines for experimental designs.

## Experimental

### Chemicals and reagents

The following chemicals were ordered from Fisher Scientific (Fair Lawn, NJ): fluorescein (FL), 5-carboxyfluorescein (5-FAM), sodium carbonate (Na_2_CO_3_), sodium bicarbonate (NaHCO_3_), sodium hydroxide (NaOH), sodium chloride (NaCl), tetrasodium salt of ethylenediaminetetraacetic acid (EDTA), sodium tetraborate, N-2-hydroxyethylpiperazine-N′-2-ethanesulfonic acid (HEPES), anhydrous acetonitrile (ACN), 4-fluoro-7-nitrobenzofurazan (NBD-F), glutamic acid (Glu), aspartic acid (Asp), ammonium glufosinate (GluF), and aminomethylphosphonic acid (AMPA). Glyphosate (GlyP) was purchased from Sigma-Aldrich (St. Louis, MO). Fused silica capillaries were ordered from Polymicro Technologies (Phoenix, AZ).

Buffer stock solutions of tetraborate, HEPES, Na_2_CO_3_, and NaHCO_3_ were each prepared at 100.0 mM in deionized water (DI water). The NBD-F stock solution at 40.0 mM was prepared in anhydrous acetonitrile. The stock solutions of fluorescein, 5-FAM, GlyP, GluF, AMPA, Glu, and Asp were individually prepared at 10 mM in DI water. The standard samples were prepared by diluting these stock solutions to the desired concentrations.

### Instrumental setup and experimental conditions

The lab-built flow-gated CE detection system has been described previously [24]. Briefly, excitation was provided by a Cobolt laser at 491 nm filtered through an interference bandpass filter at 491 ± 5 nm. A 100× oil-immersion objective was used for both laser beam focusing and fluorescence collection. Fluorescence emission was filtered through a bandpass filter centered at 525 ± 25 nm, and then was detected by a photomultiplier tube (PMT). The current signal was pre-amplified and converted to voltages by an SR570 preamplifier. A custom LabVIEW program controlled the gating flow and applied voltages for sample injections and separation, as well as recording electropherograms.

The flow-gated CE system was interconnected with polydimethylsiloxane (PDMS) interfaces, including a flow gate and tubing connectors, as described previously [25]. The separation capillary had effective and total lengths of 10.0 cm and 17.0 cm, respectively, with an ID of 10 μm and an OD of 360 μm. A Spellman high-voltage power supply (Model CZE1000R) was modified for automatic polarity switching. The BGE flow for gating was regulated by using a three-way solenoid pinch valve (Cole-Parmer, Vernon Hills, IL, USA). For flow-gated injection, the BGE gating flow was temporarily paused to allow sample accumulation at the flow gate region. Unless otherwise stated, low-conductivity plug injection and FASI-EKS were performed at −5 kV and +5 kV, respectively. Separations were performed at −20 kV with normal polarity.

### Fluorescence imaging

Fluorescence imaging was performed by using an inverted microscope (Nikon Eclipse TS100) equipped with a Mira Light Engine (Lumencor, Beaverton, OR, USA) and an AmScope CMOS camera (AF202, Irvine, CA, USA). To improve visual clarity during imaging, the original 10 μm ID separation capillary was replaced with a 40 μm ID capillary. Approximately 10 mm polyimide coating near the capillary inlet was removed by flame burning to allow optical access. The flow gate assembly was secured onto the microscope stage with adhesive magic tape. A 4× Olympus objective (Model E A4, 0.10 NA) was employed to direct cyan light from the Mira Light Engine onto the flow gate region and the capillary, and to collect emitted fluorescence for imaging. Buffer conditions during imaging were consistent with those used in the electrophoretic experiments.

### Detection of glyphosate in cereal samples

Cereal and rolled oat samples were purchased from local grocery stores. The sample processing and extraction followed the reference, where 85% recovery was obtained [26]. Briefly, each sample was ground into a fine powder, and 3 g of the powder was extracted in 25.0 mL of DI water using ultrasonication (Branson Ultrasonics, CT, USA) for 10 min. The extract was then centrifuged at 5000 RPM for 10 min (Beckman Coulter, CA, USA), and the supernatant was collected. The remaining precipitate was re-extracted with an additional 25 mL of DI water under the same conditions. The two supernatants were combined for analysis.

To perform fluorogenic derivatization, the filtered extract (through a 0.45-μm syringe filter) was diluted by 2 folds with DI water to reduce its inherent conductivity. Fifty microliters of this sample was mixed with 10.0 μL each of Na_2_CO_3_ buffer (10.0 mM), EDTA (10 mM), ACN, DI water, and NBD-F (40 mM), obtaining a final volume of 100.0 μL. For the standard addition samples, 10.0 μL of DI water was replaced with 10 μL of an aqueous standard solution containing AMPA and GlyP. Derivatization was conducted at room temperature in the dark for 30 min.

The resulting sample solutions were loaded into two 50-μL syringes, respectively, to perform alternate separations on the flow-gated CE system, following the operational protocols described before [23, 27]. The alternate sample flows were manually switched by using a 6-port valve (Vici Valco). To verify the accuracy of the one-point standard addition method, four-point standard addition was also performed for the cereal sample, Frosted Flakes.

## Results and discussion

### Simultaneous EKS

**Fig. 1:**
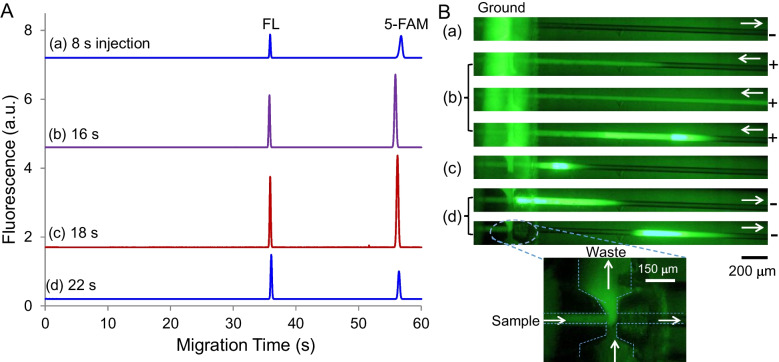
(**A**) Typical electropherograms under various FASI-EKS times as indicated in the figure. Refer to the text for experimental conditions. (**B**) Fluorescence images showing the simultaneous FASI-EKS process. (**a**) Low-conductivity plug injection. (**b**) FASI-EKS with moving boundary. (**c**) Polarity reversal. (**d**) Separation. The Insert shows the cross region. Fluorescein at 10 μM was dissolved in 1.0-mM HEPES at pH 9.5, and BGE was 40-mM Na_2_CO_3_. Arrows indicate flow directions

In the simultaneous EKS mode, BGE contains a high-mobility anion as LE, and the sample includes a low-mobility anion as TE. In this study, 40-mM Na_2_CO_3_ at pH 11.3 was used as BGE, while 1.0-mM HEPES was employed as TE in the sample. The sample pH was adjusted to 9.5 with 100-mM NaOH. A voltage of −5.0 kV at the normal polarity was applied for 8 s to introduce a low-conductivity plug, followed by FASI-EKS under the reversed polarity (5 kV) for varying durations. As can be seen in Fig. 1A, a longer FASI-EKS time led to higher peaks due to continued FASI and tITP, for which ${\text{CO}}_{3}^{2-}$ acted as LE, while HEPES^−^ served as TE. However, further increases in FASI time beyond an optimal point resulted in decreased peak heights. The signal enhancement showed approximately 3200 folds for FL and 5000 folds for 5-FAM under the conditions of Fig. 1A(c) with acceptable precision (approximately 10% RSD). The peak height decrease was attributed to the EOF pumping of the stacked analyte zone out of the capillary, as the overall EOF was greater than the electrophoretic velocities of both the LE and the analytes. Moreover, the extent of the pushout varied, leading to inconsistent amounts of analytes remaining in the capillary for separation. Given the complexity of the buffer systems involved, it was impractical to maintain a stable and reproducible EOF, especially due to the successive use of the capillary without reconditioning between runs, as is typically performed in conventional CE.

To visualize analyte migration, the FASI-EKS process was examined by using fluorescence imaging. A series of images is shown in Fig. 1B. As can be seen, during the sample medium injection, negatively charged fluorescent dyes (FL^2−^) were excluded from entering the capillary, resulting in no visible fluorescence signal within the separation channel (Fig. 1B(a)). Once the voltage polarity was reversed, the fast-moving dye anions were electrophoretically injected and concentrated at the buffer boundary through FASI (Fig. 1B(b)). Since the present buffer conditions failed to establish a stationary LE boundary as discussed in Reference [7], the EOF carried the concentrated dyes backward toward the inlet while the bright plug was continually broadening. Finally, an applied voltage at normal polarity pumped the analyte plug downstream for separation (Fig. 1B(d)). By comparing Fig. 1B(d) with Fig. 1B(c), we can see that bright dyes shifted to the rear from the front of the sample plug, resulting in a brighter rear zone. This dye shift was attributed to the rapid migration of the dye anions under the high, reversed electric field.

This re-concentration process following FASI-EKS involved reversed tITP due to the change in voltage polarity. Under these conditions, anionic analytes migrated across the TE zone and got re-concentrated at the rear boundary via tITP. The efficiency of this re-stacking process depended on the length of the sample plug. A narrow plug allowed rapid and effective re-concentration, whereas a wide plug required more time to complete the process. However, carbonate anions from the front BGE zone could interfere by trapping some of the concentrated analytes, thus hindering their re-stacking. This situation was more likely to occur when the FASI-tITP boundary formed farther away from the capillary inlet. For analytes with slow electrophoretic velocities compared to EOF, a polarity reversal following the FASI-EKS step was necessary to facilitate their migration toward the detector downstream. For improved sample concentration using simultaneous EKS, the same polarity should be maintained for the following stacking and separation [28, 29].

**Fig. 2:**
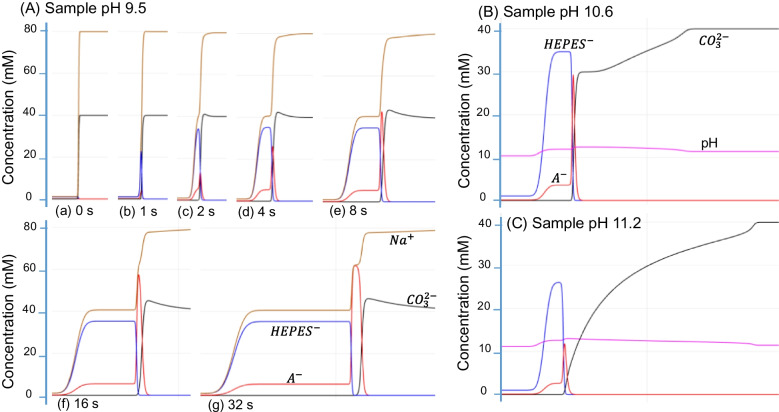
Concentration profiles of associated ions by simulation. BGE was 40-mM Na_2_CO_3_ at pH 11.3. The sample was composed of 1.0 mM HEPES and 0.10 mM acetic acid at (**A**) pH 9.50, profiles in a time lapsing sequence; (**B**) pH 10.60, at lapsing time 4.3 s; and (**C**) pH 11.22, at lapsing time 3.5 s. Sample pH was adjusted by setting appropriate Na^+^ concentrations (note: Na.^+^ indicates NaOH in Simul 6)

To illustrate the concentration profiles of associated ions, the simultaneous EKS process was simulated by using the Simul 6 program [31]. The BGE consisted of ${\text{CO}}_{3}^{2-}$ at 40 mM and Na^+^ at 80 mM with pH 10.3, while the sample solution contained 0.1 mM acetic acid (A^−^) and 1.0 mM HEPES, with the pH adjusted to 9.5. The mobilities of associated ions are listed in Table 1. The capillary (9.00 cm long and 10 μm ID) was filled with the sample solution and BGE at 1:14 ratio (with the sample plug taking 1/15 of the total volume). A voltage of 200 V, with reversed polarity, was applied across the capillary to simulate the FASI-EKS process. The concentration profiles of Na^+^, ${\text{CO}}_{3}^{2-}$, HEPES^−^, and acetate (A^−^, i.e., CH_3_COO^−^) were recorded as shown in Fig. 2A.

**Table 1:** Electrophoretic mobilities of anions used in experiments or in Simul 6 simulation

Buffer anions	Cl^−^	${\text{CO}}_{3}^{2-}$	${\text{HCO}}_{3}^{-}$	${\text{B}(\text{OH})}_{4}^{-}$	HEPES^−^	
Mobilities (× 10^–4^ cm^2^/Vs)	−6.12	−4.54	N/A	−2.58	−1.58	
Analyte anions	FL^2−^	5-FAM^3−^	NBD-GlyP^3−^	NBD-AMPA^2−^	NBD-GluF^2−^	A^−^
Mobilities (× 10^–4^ cm^2^/Vs)	−2.58	−3.40	−3.86	−3.53	−3.28	−3.12

Mobilities of FL and 5-FAM were determined in 40 mM tetraborate buffer at pH 9.20, NBD derivatives’ mobilities were determined in 20 mM tetraborate pH 10.0, and buffer anions were from Reference [30]

As can be seen, anions such as HEPES^−^ and A^−^ were quickly injected into the capillary and concentrated at the boundary, where ${\text{CO}}_{3}^{2-}$ anions served as LE and the concentrated HEPES^−^ functioned as TE. Although HEPES was initially present at only 1.0 mM in the sample, its concentration at the stacking boundary was quickly increased to a plateau value of 35 mM, which was broadening over time. For illustrative purposes, the acetic acid (A^−^) concentration in the sample was set at 0.1 mM, and the peak concentration increased with time until eventually reaching a plateau of 62 mM. In practical scenarios, however, the analyte concentrations are typically much lower, such as in a nanomolar or micromolar range, so such a plateau would rarely form in a short time. This fact allows further increase of the analyte concentration under an extended period of FASI-EKS [10].

In addition to the concentrated region of analyte A^−^ at the boundary between the LE and TE, a rear A^−^ shoulder was observed trailing the main peak, broadening toward the capillary inlet along with the HEPES plateau and their counter ion Na^+^ plateau. The continuous broadening of anionic plateaus was contributed by the rapid FASI supply of anions, but only a small portion of A^−^ was used to build the analyte region defined by LE and TE via tITP. This anionic analyte shoulder was also observed in the fluorescence images, as shown in Fig. 1B(b). Additionally, the simulated ion profiles provided insights about the conductivity difference between the sample and the BGE regions. The lower conductivity in the sample zone resulted in a higher electric field, as illustrated in Fig. 2B(g). It should be noted that the practical protocol involved EOF, while the simulation ignored the EOF pumping. Consequently, a portion of the concentrated shoulder might be pumped out of the capillary before the polarity reversal for separation. This would lead to analyte loss and a corresponding decrease in the peak sizes, as observed in Fig. 1A(d).

**Fig. 3:**
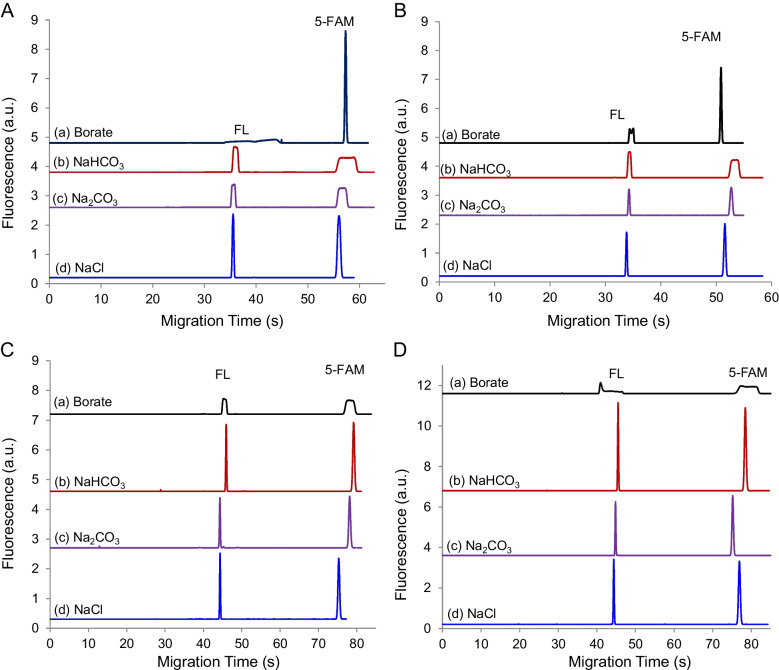
Typical electropherograms obtained under various pairs of LE/TE. (**A**) BGE 40.0 mM Na_2_CO_3_ at pH 11.3. (**B**) BGE 60.0 mM NaHCO_3_ at pH 9.0. (**C**) BGE 20 mM tetraborate at pH 10.0. (**D**) BGE 100 mM HEPES at pH 8.2. Chemicals a–d were each added to sample solutions at 1.0 mM

When sample buffer pH was increased, for example, to pH 10.60, the influence by hydroxide (OH^−^, ~ 0.40 mM) became significant. Due to their higher electrophoretic mobility compared to carbonate anions, concentrated OH^−^ plug began to penetrate into the carbonate region, resulting in a local increase in pH, as illustrated in Fig. 2B. To maintain charge neutrality, this OH^−^ intrusion led to a corresponding decrease in carbonate ion concentrations. The extent of this penetration became even more pronounced at higher sample pH levels such as pH 11.22 (OH^−^ at 1.6 mM, Fig. 3C), where concentrated OH^−^ zone expanded further, weakening the function of carbonate anions as LE. Notably, the reduced analyte A^−^ peak in Fig. 2C compared to Fig. 2B was caused by the higher conductivity at the higher pH.

### Sequential EKS

In the sequential EKS setup, BGE contains an anion with low electrophoretic mobility, while the sample solution includes an anion with a high mobility, as demonstrated previously [23]. Anions in the sample can be injected and concentrated via FASI, following electroosmotic introduction of a low-conductivity plug. To evaluate concentrating efficiency under different conditions, a series of BGEs including Na_2_CO_3_, NaHCO_3_, borate, and HEPES was evaluated by using sample solutions containing various anions, as shown in Fig. 3. The electrophoretic mobilities of the relevant ions are listed in Table 1.

These electropherograms suggest that, for effective FASI-tITP focusing based on sequential EKS, the electrophoretic mobility of anions in the sample should be greater than that of both analyte anions and BGE anions. Notably, the results in Fig. 3A(d) and 3B(d) also indicate that analyte mobilities do not necessarily need to be greater than those of the BGE anions to achieve sharp analyte peaks, although a smaller mobility of BGE ions could improve focusing results, as shown in Fig. 3C(d) and 3D(d). The exceptions of Fig. 3A(a) and 3B(a) should be considered as simultaneous EKS for 5-FAM, while the mobility of fluorescein (FL) fails to meet the criteria of both sequential and simultaneous EKS, since FL mobility was comparable to borate’s (B(OH)_4_^−^, see Table 1). The flat peaks in Fig. 3 resulted from the fact that the mobility of anions in BGE was equal to or greater than that of anions in the sample, while both exceeded the mobilities of analytes.

**Fig. 4:**
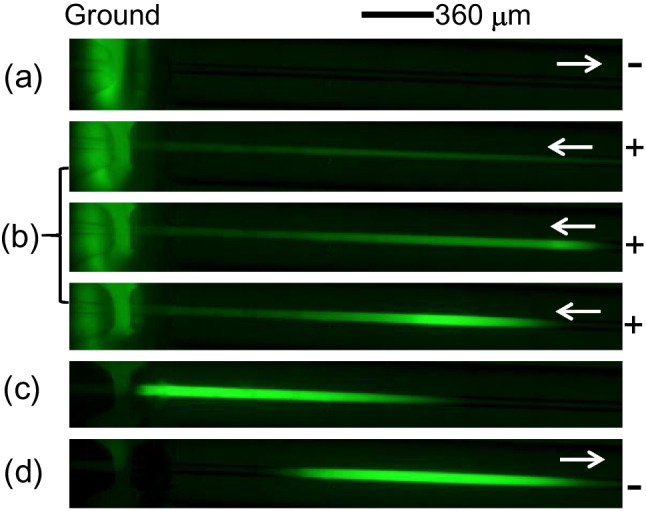
Sequential images of FASI followed by tITP. (**a**) Sample media injection. (**b**) FASI. (**c**) Polarity reversal. (**d**) Separation. Sample consisted of 2.0-μM 5-FAM and 1.0-mM carbonate at pH 11.3. Normal-polarity or reversed-polarity voltage was applied across the capillary at 2.5 kV for 3.0 s or 3.5 s, respectively

To visualize the FASI process, fluorescence imaging was performed, and the sequential images are shown in Fig. 4. As can be seen, the FASI step electrophoretically injected and concentrated anionic 5-FAM at the conductivity boundary, forming a bright dye band that expanded over time (see Fig. 4b). This expansion came from the continuous accumulation of dye anions along the capillary injected through FASI. Simultaneously, the EOF carried the dye band toward the inlet, gradually pumping out portions of the low-conductivity plug until the voltage was reversed to conduct separation. Figure 4c and d show a wide dye plug; without further focusing, flat top peaks are expected like those in Fig. 3. Finally, the broadened dye band was transported downstream by EOF, beyond the region observable by fluorescence imaging, where further processing of the dye plug occurred.

**Fig. 5:**
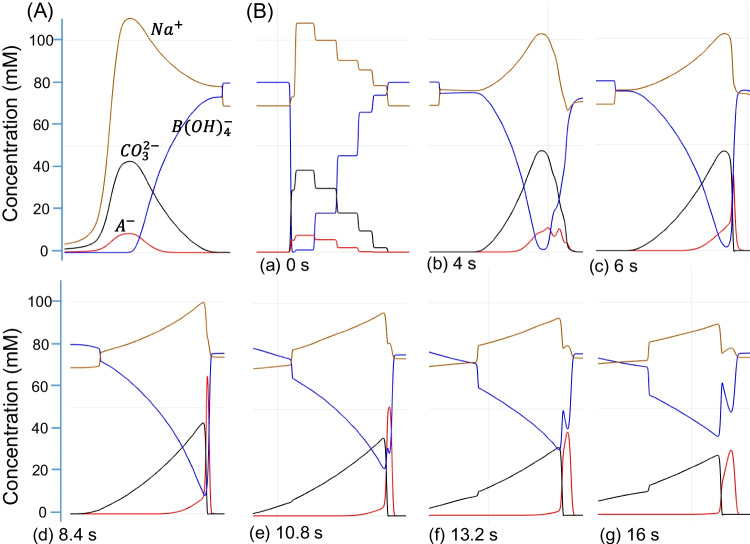
Sequential ion concentration profiles simulated by using Simul 6. (**A**) Profiles at the end of FASI. (**B**) Profiles with the tITP progress in a time sequence. Note that (B(a)) is the initial equivalent profile of (A) after FASI. The sample consisted of 1.0-mM Na_2_CO_3_ and 0.2 mM acetic acid (A^−^) at pH 9.5, and BGE was 80 mM borate at pH 10.0

To investigate the behavior of ions following FASI, concentration profiles were simulated by using Simul 6, as shown in Fig. 5. The resulting ion concentration profiles at the end of FASI are presented in Fig. 5A. During FASI, both carbonate and analyte anions were simultaneously injected and concentrated at the buffer boundary, with carbonate reaching a peak concentration of approximately 42 mM and the analyte A^−^ reaching 8.8 mM.

**Fig. 6:**
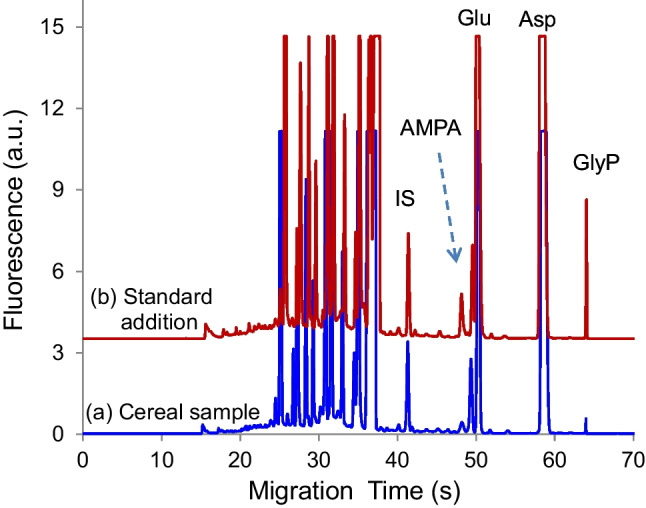
Typical electropherograms of a cereal sample (Frosted Flakes) and its standard addition. The supernatant was diluted by 2 folds. Standard additions of AMPA and GlyP were 100 nM and 200 nM, respectively. Internal standard (IS) was 250 nM GluF

Due to the limitations of the Simul 6 program, an equivalent ion profile (Fig. 5B(a)) was manually set as the starting condition to simulate the ion behaviors under a normal-polarity voltage. The simulated results (Fig. 5B) indicate that carbonate and analyte anions migrated backward simultaneously under the applied voltage. With further changes of ion profiles, carbonate anions began to serve as LE (see Fig. 4B(c)), while borate anions functioned as TE, enabling the tITP focusing of A^−^ into a sharp peak (Fig. 5B(d)). However, the sharpness of this peak diminished once the front and the rear borate buffer zones merged into a relatively continuous system, weakening the TE functionality of the borate zone (Fig. 6B(e)). Along with the decreasing concentration of the carbonate plug, this led to the broadening of the analyte plug until the two zones became fully separated. From this moment, the separation of analytes was totally based on capillary zone electrophoresis.

### Detection of herbicide residues in cereal samples using sequential EKS

The sequential EKS method has previously been used to measure herbicide residues in surface water samples [23]. In this study, we employed a carbonate–borate pair as LE/TE to preconcentrate glyphosate (GlyP) and its major degradation product aminomethylphosphonic acid (AMPA) in cereal samples.

BGE conditions were optimized to include 20 mM tetraborate at pH 10 as the separation buffer and 1.0 mM carbonate at pH 10.3 as the sample buffer. The timing parameters were set to 5 kV × 6 s for low-conductivity media injection, followed by −5 kV × 7 s for FASI. The tITP and subsequent separation were conducted at −20 kV. Method validation showed good linearity, reproducibility, and limits of detection, consistent with our previous results [23]. Under these optimized conditions, the detection sensitivity was enhanced by 947, 591, and 718 folds for GlyP, AMPA, and GluF, respectively, compared to normal injection at 5.0 kV × 0.5 s, with the intra-day or inter-day reproducibility below 4.0%. These results demonstrate improvement in signal enhancement for all three analytes compared to the previous protocol [23], which was attributed to the effectiveness of the present LE/TE pair.

The electrophoretic mobilities of NBD-F derivatives of GluF, AMPA, and GlyP were determined by using BODIPY as a neutral marker to be −3.28 × 10^–4^, −3.53 × 10^–4^, and −3.86 × 10^–4^ cm^2^V^−1^ s^−1^, respectively, in 20 mM tetraborate buffer at pH 10.0. These values fall between the mobilities of carbonate and borate anions, as shown in Table 1. To achieve baseline separation of the three derivatives from interferences including glutamate, aspartate, and the hydrolytic product of NBD-F, the pH of the separation buffer was optimized to pH 10, as the effective charge of each analyte is pH-dependent [23].

Finally, the analytical protocol was applied to determine GlyP and AMPA residues in five commercial cereals and one rolled oat sample. Typical electropherograms are demonstrated in Fig. 5. GluF was added as an internal standard (IS) considering no GluF was detected in the cereal samples. EDTA at 1.0 mM was added to the derivatization mixture to remove metal cations. The results are summarized in Table 2. As shown, corn cereals contained small amounts of both GlyP and AMPA, indicating traces remained even after food processing. In contrast, neither compound was detected in wheat cereals. The rolled oats also contained slightly higher levels of both residues compared to corn cereals, possibly due to the minimal processing typically applied to rolled oats.

**Table 2:** Herbicide residues determined in cereal and oat samples

Samples	Ingredient	ng/g ± SD, *n* = 3	
		GlyP	AMPA
Corn squares	Milled corn	301 ± 6	363 ± 8
Frosted Flakes	Milled corn	142 ± 7	190 ± 4
Rolled oats	Oat	437 ± 9	484 ± 7
Bran flakes	WG wheat	ND	ND
Shredded wheat	WG wheat	ND	ND
Frosted shredded wheat	WG wheat	ND	ND

*WG*, whole grain; *ND*, not detected

These results suggest that herbicides containing GlyP were likely used on corn and oat crops, either for weed control or as a desiccant before harvest. In the case of wheat, the absence of detectable GlyP residues could be due to one or more factors: GlyP might not be applied to the crop, it might not accumulate in wheat grains, or it might have degraded during food processing.

## Conclusions

The working principles of the FASI-EKS were investigated by using electropherograms of model analytes under various combinations of LE/TE, fluorescence imaging, and computer simulations. These complementary approaches were intended to provide a holistic understanding of the FASI-EKS process, thereby facilitating its practical applications. To highlight differences in the EKS mechanisms, two operational modes were examined: simultaneous EKS and sequential EKS. In the simultaneous EKS mode, FASI and tITP occurred concurrently, whereas in the sequential EKS, FASI was performed first, followed by tITP.

The simultaneous EKS demonstrated significant analyte preconcentration when a steady boundary was maintained [7]. However, reproducibility was a concern since consistently positioning the boundary at the same location across runs proved challenging. This boundary location was critical because it directly influenced the amount of analytes injected into the capillary due to the FASI amplification. Additionally, the analyte plugs concentrated by tITP were further re-concentrated at the rear boundary under the reversed polarity from the FASI voltage. This secondary concentration step could be incomplete due to the sweeping of original LE anions.

In contrast, the sequential EKS provided lower concentration enhancement compared to the simultaneous mode but offered improved control over experimental conditions. As a result, it yielded more reproducible peaks, thus enhancing accuracy and precision in quantitative analysis. Given these advantages, the sequential EKS was employed for detecting GlyP and AMPA residues in cereal samples using the flow-gated CE system. The results validated the method’s feasibility for practical applications.

## Supplementary Information

Below is the link to the electronic supplementary material.

Supplementary Material 1 (MP4 3.28 MB)

Supplementary Material 2 (MP4 3.10 MB)

## Data Availability

Data available upon request to the corresponding author.
